# Rehabilitation of discourse impairments after acquired brain injury

**DOI:** 10.1590/S1980-57642014DN81000009

**Published:** 2014

**Authors:** Gigiane Gindri, Karina Carlesso Pagliarin, Fabíola Schwengber Casarin, Laura Damiani Branco, Perrine Ferré, Yves Joanette, Rochele Paz Fonseca

**Affiliations:** 1 Postgraduate Psychology Program – Department of Psychology – Pontifical Catholic University of Rio Grande do Sul, Porto Alegre, Rio Grande do Sul, Brazil.; 2 Université de Montréal, Montréal, Canada.

**Keywords:** brain injuries, communication, language, rehabilitation

## Abstract

Language impairments in patients with acquired brain injury can have a negative impact on social life as well as on other cognitive domains. Discourse impairments are among the most commonly reported communication deficits among patients with acquired brain damage. Despite advances in the development of diagnostic tools for detecting such impairments, few studies have investigated interventions to rehabilitate patients presenting with these conditions.

**Objective:**

The aim of this study was to present a systematic review of the methods used in the rehabilitation of discourse following acquired brain injury.

**Methods:**

The PubMed database was searched for articles using the following keywords: "rehabilitation", "neurological injury", "communication" and "discursive abilities".

**Results:**

A total of 162 abstracts were found, but only seven of these met criteria for inclusion in the review. Four studies involved samples of individuals with aphasia whereas three studies recruited samples of individuals with traumatic brain injury.

**Conclusion:**

All but one article found that patient performance improved following participation in a discourse rehabilitation program.

## Abstract (pt)

Os prejuízos de linguagem observados em pacientes com lesões cerebrais adquiridas podem ocasionar impacto negativo na vida social do indivíduo assim como em outros domínios cognitivos. Prejuízos em habilidades discursivas estão entre os déficits comunicativos mais comumente reportados em pacientes com lesão cerebral adquirida. Apesar do avanço dos estudos sobre instrumentos para diagnosticar tais déficits, ainda são escassas as investigações que proponham um programa de intervenção para sequelas discursivas.

**Objetivo:**

Assim, esta revisão sistemática visou a caracterizar estudos de reabilitação do processamento comunicativo discursivo em casos pós-lesão cerebral adquirida.

**Métodos:**

Na base de dados PubMed utilizaram-se palavras-chave dos construtos "reabilitação", "lesão neurológica", "comunicação" e "habilidades discursivas".

**Resultados:**

Foram encontrados 162 abstracts, mas apenas sete apresentaram os critérios de inclusão para esta revisão. Quatro estudos envolveram amostras com indivíduos afásicos e três com indivíduos com traumatismo cranioencefálico.

**Conclusão:**

Todos, com exceção de um artigo, verificaram evolução dos pacientes após intervenção baseada no discurso.

## INTRODUCTION

Motor and cognitive impairments are common consequences of acquired brain injury, and are often observed after strokes or traumatic brain injury (TBI). Such impairments may have a significant impact on the social functioning and quality of life of patients and their caregivers.^1,2^ Although many cognitive processes may be influenced by acquired brain injury, language complaints are among the most frequently reported by patients and their families.^3^ Language impairments may also have a negative influence on cognitive domains such as memory, attention and executive functions, as most of these processes are mediated by language.^4,5^

Both of the cerebral hemispheres play an important role in language processing. The left hemisphere (LH) is more closely associated with the formal components of language, such as phonology, syntax, semantic and morphology.^6-9^ The right hemisphere (RH), on the other hand, is more heavily involved in pragmatic, lexical-semantic, prosodic and discursive processing.^10-12^

Although discourse processing deficits are an important cause of functional impairment, they have been scarcely studied in the literature. "Discourse" consists of using spoken or written language to convey ideas in an organized manner, and involves varying levels of language representation and semantic processing;^13^ Discourse relies on linguistic skills for grammatical processing, on pragmatic reasoning to understand the communicative intentions of other speakers and for inferential processing, and also recruits cognitive abilities such as attention, memory and executive functions.^14^ As a result, discourse is generally considered the most complex of communicative abilities.^15^ Van Djik notes that discourse is a way of representing the world through different levels and^16^ structures of language (words, phrases, sentences, speeches) in interaction with cognitive information processing.

Studies have shown that patients with acquired brain injury can have difficulty integrating the elements of a story into a coherent whole so as to comprehend it. These individuals may also have trouble taking listener needs into account^12^ and understanding the intentions of other speakers.^17^ Discourse production deficits have also been reported in the literature, the most common being impairments in storytelling,^18^ tangential speech, or difficulty staying on topic,^19,20^ and problems with conversational turn-taking.^21,22^

Some studies, such as the systematic review conducted by Ferré, Ska, Lajoie, Bleau and Joanette,^23^ have posited that both discourse production and comprehension require the involvement of both cerebral hemispheres. The LH recognizes words and engages in syntactic processing, while the RH is responsible for integrating information into a coherent whole. In addition, the RH is more heavily involved in locating and accessing less obvious semantic information.^17^

A number of authors have studied communication assessment in an effort to identify which instruments would be most sensitive and specific for clinical diagnoses and for the planning of therapeutic interventions. While most of these studies sought to investigate the role of both hemispheres, some have focused more specifically on the RH.^24-27^ Most discourse assessment batteries assess oral narrative ability through storytelling involving characters (spontaneous production) and investigate conversational discourse through the comprehension and production of dialogue between two or more speakers.^28,29^

Although research has been conducted into discourse impairments in individuals with brain injury (especially those with unilateral lesions), and assessment instruments are available to detect these conditions, very few studies have described methods for treating communicative deficits. Therefore, such methods have not been replicated and their effectiveness has not been assessed. Recent literature has demonstrated a growing interest in evidence-based rehabilitation and over the past few years some systematic reviews have attempted to establish guidelines for cognitive interventions.^30-32^ Reviews such as those by Ferré et al.^23^ and Tompkins^33^ have also sought to identify cognitive abilities that may have been neglected by rehabilitation research (see, for instance,^23,33^). These investigations have highlighted inconsistencies in studies of communication rehabilitation, especially those that target conversational discourse.^34^

In an attempt to assess the current state of research on communication impairments after acquired brain injury, the aim of the present study was to conduct a systematic review of the literature on discourse processing in individuals with such conditions.

## METHODS

The PubMed database was searched in January of 2014 for articles investigating the following four constructs: "rehabilitation" AND "brain damage" AND "communication" AND "discourse abilities." Articles were retrieved using keywords that are frequently used in the literature on these topics. The keywords used for each construct topic were as follows:

[a] Rehabilitation: "rehabilitation" OR "treatment" OR "functional recovery" OR "readaptation" OR "reeducation", "training" OR "intervention" OR "therapy" OR "remediation";
[b] Brain damage: "right hemisphere damage" OR "left hemisphere damage" OR "stroke" OR "lesion studies" OR "cerebrovascular disease" OR "cerebrovascular accident" OR "brain injury" OR "brain damage" OR "traumatic brain injury" OR "closed head injury";
[c] Communication: "linguistic" OR "language" OR "aphasia" OR "communication" OR "communicative";
[d] Discourse abilities: "discourse" OR "narrative" OR "conversation" OR "conversational" OR "dialogue".

The electronic search was performed in two steps. Firstly, the sets of keywords referring to each of the four constructs were entered separately in the search box. Then, the results of all four searches were combined, and hand-filtered by the researchers. This review focused on articles published in English, French, Spanish or Portuguese.

All abstracts were independently screened by two reviewers, and articles were included in the review if they fulfilled the following criteria:

[a] comprising an empirical study,
[b] containing at least one adult with acquired brain injury in the sample,
[c] focusing on discourse rehabilitation for patients with acquired brain injury,
[d] providing a description of the intervention used,
[e] involving non-pharmacological interventions.

**Figure 1:**
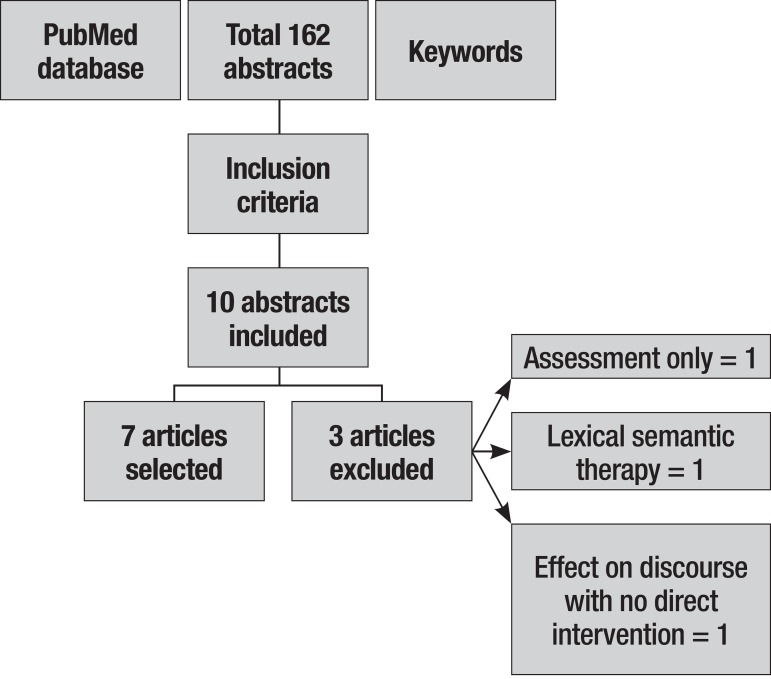
Flow of articles through the systematic review process.

When reviewers did not agree on article inclusion, a third rater read the article and made the final decision. The flow of articles through the systematic review process is illustrated in Figure 1.

## RESULTS

Table 1 describes the studies included in the review. The characteristics of participants in the studies, the aim of each article, the pre- and post-intervention assessment method, and the results of each study are also given in the table.

**Table 1:** Description of studies reviewed.

Study	Journal	Objective	Sample	Assessments		Details of the intervention	Results
				Timepoints of assessment	Instruments used		
Cannizzaro & Coelho (2002)	Brain injury	To investigate the treatment of discourse production deficits, specifically story grammar ability, in an individual with traumatic brain injury (TBI).			- Screenings of hearing and visual acuity and visual perception- Rancho Los Amigos Levels of Cognitive Functioning- Galveston Orientation and Amnesia Test- Dementia Rating Scale- WAB	Participants were presented with five colour prints of Rockwell paintings. The subjects were instructed to tell a story about the Picture. The stories were elicited from the patient on three occasions: at baseline, and then on a weekly basis, as a treatment probe. Three 60-minute treatment sessions were conducted each week (3 weeks). After the baseline phase, two treatment conditions were introduced: “story retelling” and “story generation”. There was a 1-week break in treatment between the “retelling” and “generation” conditions.	Story grammar ability initially appeared to have been effective. The patient had good performance during the treatment but follow-up probes indicated that the effects of treatment were not maintained.
Cherney & Halper (2008)^40^	Topics in Stroke Rehabilitation	To describe the treatment of adult patients with aphasia and cognitive impairment using conversation training software.	n=2 adults, one with nonfluent aphasia after ischemic stroke, and one with fluent aphasia following hemorrhagic stroke		- WAB- Raven's Progressive Matrices- MMSE (Mini-Mental State Examination)- Quality of Communication Life Scale- CADL-2 (Communication Activities of Daily Living-2)- Communicative Effectiveness Index	Three routines were developed for each participant, and individuals were required to practice these at home using a software program (AphasiaScripts^TM^) over the course of three weeks (30min/day). Routines involved dialogues and monologues. The therapist met the patient once a week for 30 min. All material was recorded and transcribed for analysis.	Improvements in the content and grammatical complexity of the conversation routines were observed in all participants.
Cherney, Halper, Holland, & Cole (2008)^41^	American Journal of Speech-Language Pathology	To describe a computerized rehabilitation program for conversation training, and test its use in adult patients with aphasia.	n=3 adults with chronic aphasia (Broca's, Wernicke's and Anomic)		- WAB- CADL-2 - Quality of Communication Life Scale	Same as Cherney et al., 2008.	Positive changes were observed in the content, grammar and conversation routines used by participants.
Manheim, Halper & Cherney (2009)^42^	Archives of Physical Medicine and Rehabilitation	To assess changes in the communication performance of patients who took part in a computerized conversation rehabilitation program.	n=20 adults with chronic aphasia		- Communication Difficulty subscale of the Burden of Stroke Scale (BOSS)	Speech therapy sessions were conducted to develop relevant conversation routines for the patient. Participants also had weekly appointments with the therapist, and underwent 9 weeks of daily training (30 min/day) using the computer software. Three stages of practice: (1) patient observes the routine with verbal and visual input; (2) repetition of sentences in the conversation; and (5) conversation practice with a virtual therapist.	There were no significant differences between pre-treatment and baseline assessments, but significant improvements in communication were detected in post-treatment and follow up assessments .
Sim, Powe & Togher (2013)	Brain Injury	To describe conversational discourse change after joint communication training for individuals with TBI and their everyday communication partners (ECPs), as compared to untrained controls.	n=14 adults with severe TBI each paired with an ECP and control group of 15 participants with severe TBI and their paired ECP		- WAIS-III- Global Ratings of Quality of Conversations- Adapted Measure of Participation in Conversation	Participants in the JOINT group received social communication training weekly over 10 weeks (2.5 hour weekly group session and a 1 hour weekly individual session). The CONTROL group did not receive any training until post-training measures were completed.The aim was to maximize communicative effectiveness using several behavioural techniques (e.g. role- plays, video feedback, cues for self-monitoring), participants were trained to apply appropriate verbal and non-verbal behaviour in social situations. ECPs were trained to support their partner with TBI to contribute more successfully in conversations, by using positive collaborative and production strategies to provide appropriate feedback and scaffolding in everyday casual conversations. In individual sessions, goals were set and participants were guided in problem-solving or troubleshooting the use of new strategies	JOINT training resulted in greater changes in discourse behaviour for individuals with TBI, compared to a control group which did not receive training
Togher et al., 2013	Journal of Rehabilitation Medicine	To determine effectiveness of communication training for partners of individuals with severe TBI.	n=44 adults with moderate-severe TBI(14 JOINT group, 15 TBI SOLO group, 15 Control group)		- Wechsler Memory Scale- WAIS-III- Controlled Oral Word Association Test- Scales of Cognitive Ability for Traumatic Brain Injury	Same as Sim et al. (2013)	Training communication partners (JOINT group) was more efficacious in improving everyday interactions of individuals with TBI than training the person with TBI alone (TBI SOLO group).
Whitworth, 2010^29^	Seminars in Speech and Language	To investigate the efficacy of a rehabilitation protocol which combines different levels of language complexity (word, sentence, and narrative).	n=2 cases, one adult with nonfluent aphasia, one adult with fluent aphasia, and n=23 neurologically healthy controls		-- Pyramids and Palm Trees Test- Kissing and dancing test- The Object Action Naming Battery- Verbs and Sentences test- Cinderella narrative	Therapy sessions were conducted twice a week over the course of 10 weeks (metalinguistic model). Picture sequences were used as prompts for the narrative. Focus was on the identification of main events and correct naming of the verbs and nouns depicted, so as to create an argumentative structure. A grammar-based narrative discourse structure was introduced, and sentences with a beginning, middle and end were created to narrate the story.	The integration of the narrative structure with words and sentences helped improve communication in daily life.

## DISCUSSION

A number of studies in the literature have tested language rehabilitation programs in individuals with acquired brain injury.^35,36^ However, these studies have focused mostly on therapy for anomia. The aim of the present study was to review the literature on discourse rehabilitation in adult patients with brain damage. although only seven studies met the initial inclusion criteria, as can be observed in Figure 1.

Most of the studies retrieved in the original search focused on language rehabilitation in patients with aphasia. This could be due to the fact that communication impairment following unilateral LH damage is a common cause of aphasia, which is, in turn, the most prevalent acquired language impairment.^37^ Therefore, the treatment of aphasia is a major goal of rehabilitation studies.^35^ However, even among these patients, discourse rehabilitation has been poorly investigated.^31^ Another possible cause of the lack of research on discourse rehabilitation following acquired brain injury is the fact that communication impairments following RH damage are underdiagnosed, and consequently, undertreated.^38^ The scarcity of research into discourse impairment among populations with bilateral or RH damage precludes the development of structured rehabilitation programs for patients with these conditions.

Three articles described rehabilitation programs aimed at individuals with TBI. These studies found that the cognitive impairment exhibited by these patients, who displayed attentional, mnemonic and executive alterations, led to difficulties in linguistic processing as well as discourse impairment. These findings underscore the need for further research into rehabilitation programs for patients with acquired brain injury.^39,40^

In spite of advances in communication assessment, few instruments have been developed to assess the different types of discourse, such as autobiographical and procedural. Even though studies show that such impairments may have a significant impact on the social functioning of individuals with brain damage, studies aimed specifically at discourse rehabilitation are still lacking, as are investigations of the efficacy of these interventions.^23,33,41^

Three of the studies included in the present review were conducted by the same group of researchers,^42-44^ who developed a software program for discourse rehabilitation. Their studies showed improvements in the communication skills of individuals with aphasia who completed the treatment program. Although the therapist was not an active participant in the rehabilitation process, patients contributed significantly to the development of the programs, so as to ensure that their needs were addressed.^43^ The use of a computer appeared to make rehabilitation more accessible to the patients, who were able to complete the activities in their own home. These methods have the added advantage of allowing participants to engage in rehabilitation activities multiple times a week.^30^ However, there may be some drawbacks of exclusive use of software for language rehabilitation, such as the fact that human interaction, which favors the development of communication skills, is absent from the treatment. Simmons-Mackie, Elman, Holland and Damico^45^ have suggested that the benefits of group therapy may stem from the fact that it favors the formation of communication dyads, which simulate real social interactions.

Social isolation and restriction in social interactions after stroke may have a negative impact on quality of life. Similarly, the inability to work may lead to lower quality of life, especially in individuals younger than 65 years, as employment has been shown to have an influence on self-concept, social status and social relationships^46^. Therefore, problems of social identity may be one of the factors responsible for worse subjective and psychological well-being following stroke. Other factors that may contribute to this situation are functional incapacity, cognitive deficits and depression. The low social support associated with reduced social interaction could also increase feelings of loneliness and hopelessness, and may have an impact on the efficacy of therapeutic interventions.^47^

The articles included in the present review assessed participant performance at *baseline*, immediately before and after treatment, and at follow up. Participants were evaluated using ecological assessments scales,^42-44^ language assessment batteries^29,42,43^ and story-telling.^29^

Although pre- and post-assessment measures are important, they may not be able to indicate whether specific discourse skills were acquired as a result of rehabilitation, or determine how long the treatment should continue. It may have also been useful to assess participants during the intervention period, so as to monitor patients' progress over the course of treatment. The variability in assessment methods across the studies reviewed also prevented comparisons between their results.^23,33^

It is important that studies of communication rehabilitation provide detailed descriptions of their interventions so that their methods can be replicated.^34^ Factors including variability in study design, small sample sizes, and the omission of methodological descriptions make it difficult to replicate these studies and to compare their results, preventing the generalization of findings to other populations.

Some authors have claimed that the existing literature is sufficient to provide a solid basis for the development of rehabilitation programs for patients with aphasia.^30,31^ However, there is no consensus as to the efficacy of these procedures.^48^ Three of the seven studies included in the present review had no control group and based their results on two or more cases. These studies can only provide class III evidence for the efficacy of the rehabilitation programs used. The study conducted by Whitworth^29^ was able to provide class II evidence, as it assessed the efficacy of an intervention in two clinical cases and involved the use of a control group.^30^ Studies that provide class II and III evidence may be useful in proving the efficacy of rehabilitation programs, defined as the probability an individual from a particular clinical population will benefit from the intervention.^49,50^

The current literature provides recommendations for interventions aimed at improving discourse impairments, as well as guidelines on how to reduce impairments and improve communication through realistic communication activities, especially those that involve social interaction.^23^ Although recommendations based on clinical practice may lack the methodological rigor of randomized controlled trials, or class I evidence, these methods have produced positive results in the past.^30,51^ However, studies of the effectiveness of language rehabilitation programs are insufficient to draw firm conclusions, and there is a clear need for scientifically based evidence, and for clinical trials similar to those conducted for medications and transcranial magnetic stimulation in patients with aphasia. One of the most important methodological limitations of the studies analyzed is the variability in the treatment methods used. Although in clinical practice methods must be adapted to each particular case, these variations make it difficult to identify the strengths and weaknesses of each respective method.^52^

Thus, there is a need for further research into the benefits that patients with acquired brain damage may expect to obtain from discourse-based treatments. It is also necessary to determine which methods should be used and what objectives should be pursued in rehabilitation studies, as well as what instruments should be used to assess participant performance. In conclusion, the development of rehabilitation programs for patients with discourse impairments could make significant contributions to clinical practice in speech therapy and neuropsychology.^51^ It is necessary to seek a consensus regarding general aspects of discourse rehabilitation, and to determine which instruments should be used to search for evidence of the efficacy of such interventions in improving sociocommunicative interactions and quality of life in patients with acquired brain injury and in their relatives.

The present findings should be interpreted in light of a few limitations. The keywords used in the electronic search retrieved a relatively small number of articles, which may impair the generalizability of the present results. The variability in the presentation of discourse alterations following acquired brain lesions may also impair the generalization of findings across samples. Consequently, the results of the articles discussed in the present review should be interpreted exclusively in the context of the populations studied. Nevertheless, the present review was able to identify the need for further research into discourse rehabilitation in populations with acquired brain injury, as well as for a greater number of publications in the area. It is suggested that similar reviews be conducted based on articles retrieved from other electronic databases, using different sets of search words. Additionally, the effectiveness of other interventions, such as rehabilitation programs aimed at the caregivers or relatives of patients with discourse impairments associated with neurological conditions, should also be investigated.
